# X-ray phase imaging: from synchrotron to hospital

**DOI:** 10.1098/rsta.2013.0023

**Published:** 2014-03-06

**Authors:** Atsushi Momose, Wataru Yashiro, Kazuhiro Kido, Junko Kiyohara, Chiho Makifuchi, Tsukasa Ito, Sumiya Nagatsuka, Chika Honda, Daiji Noda, Tadashi Hattori, Tokiko Endo, Masabumi Nagashima, Junji Tanaka

**Affiliations:** 1 Institute of Multidisciplinary Research for Advanced Materials, Tohoku University, 2-1-1 Katahira, Aoba-ku, Sendai, Miyagi 980-8577, Japan; 2 KonicaMinolta Medical and Graphic, Inc., 2970 Ishikawa-machi, Hachioji, Tokyo 192-8505, Japan; 3 Laboratory of Advanced Science and Technology for Industry, University of Hyogo, 3-1-2 Kouto, Kamigori, Hyogo 678-1205, Japan; 4 Nagoya Medical Center, 4-1-1 Sannomaru, Naka-ku, Nagoya, Aichi 460-0001, Japan; 5 Department of Anatomy, Saitama Medical University, 38 Morohongo, Moroyama, Iruma, Saitama 350-0495, Japan; 6 Department of Radiology, Saitama Medical University, 38 Morohongo, Moroyama, Iruma, Saitama 350-0495, Japan

**Keywords:** grating interferometry, phase contrast, X-ray, clinics

## Abstract

With the aim of clinical applications of X-ray phase imaging based on Talbot–Lau-type grating interferometry to joint diseases and breast cancer, machines employing a conventional X-ray generator have been developed and installed in hospitals. The machine operation especially for diagnosing rheumatoid arthritis is described, which relies on the fact that cartilage in finger joints can be depicted with a dose of several milligray. The palm of a volunteer observed with 19 s exposure (total scan time: 32 s) is reported with a depicted cartilage feature in joints. This machine is now dedicated for clinical research with patients.

## 1. Introduction

The advantage of using X-ray phase information in X-ray imaging has been attracting attention especially since the 1990s, thanks to the development of digital X-ray imaging technology and synchrotron radiation facilities [1]. While conventional X-ray imaging generates contrast relying on X-ray attenuation (absorption contrast) by an object, and its sensitivity to weakly absorbing objects is therefore poor, X-ray phase contrast imaging can visualize such an object relying on the phase shift or refraction of X-rays caused by the object. This is because the real part of the refractive index is much larger than its imaginary part, which causes absorption contrast in the hard X-ray energy region.

It should be emphasized that almost all current X-ray imaging research using X-ray phase information is based on the X-ray phase contrast and furthermore enables quantitative measurement of X-ray phase shift or refraction. The methods do not simply record a phase-contrast picture, and we refer to these methods as *X-ray phase imaging*. While absorption contrast and built-in contrast due to X-ray optical elements and their alignment are mixed and indistinguishable in a resultant phase contrast picture, X-ray phase imaging enables us to extract X-ray phase shift or refraction by the combination of X-ray phase contrast imaging and a phase measurement (or phase retrieval) method. Although multiple phase contrast pictures are normally acquired and processed by a computer in X-ray phase imaging, sophisticated understanding of an object is expected, exceeding the potential of X-ray phase contrast imaging, which simply records a phase contrast picture.

Medical applications of X-ray phase imaging methods have been studied mainly with synchrotron radiation. However, clinical applications have not been reported so far, although *phase contrast* mammography has been developed based on propagation-based contrast enhancement [2]. Because the locations and accessibility of synchrotron radiation facilities are very limited, extensive use of X-ray phase imaging for patients is not straightforward.

If the implementation of X-ray phase imaging is possible in hospitals, it will benefit radiologists and patients widely. X-ray *phase contrast* mammography has been implemented with a conventional X-ray source [3], which is available commercially. As mentioned, the promotion from X-ray *phase contrast* imaging to X-ray *phase* imaging is attractive for gaining an advantage from X-ray phase information. Since X-ray grating interferometry, such as X-ray Talbot interferometry reported in 2003 [4] and X-ray Talbot–Lau interferometry reported in 2006 [5], was proposed as a new X-ray phase imaging approach, its practical use for clinical X-ray phase imaging has been realistically expected. An X-ray grating interferometer, which consists of transmission gratings aligned along an X-ray optical axis, functions with polychromatic cone-beam X-rays. A conventional X-ray source can be used with it for phase imaging with a practicable exposure time.

Furthermore, it has been attracting attention that a third item of information relating to ultrasmall-angle scattering can be extracted from the identical phase-measurement dataset [6]. The image can be generated by calculating interference visibility in the recorded images. Although individual scatterers, such as a microcalcification and a fibre, in an object cannot be resolved, their density distribution can be visualized by this image. Thus, X-ray phase imaging provides ternary maps (i.e. absorption, refraction (differential phase) and visibility images), enabling sophisticated understanding of an object.

**Figure 1.**
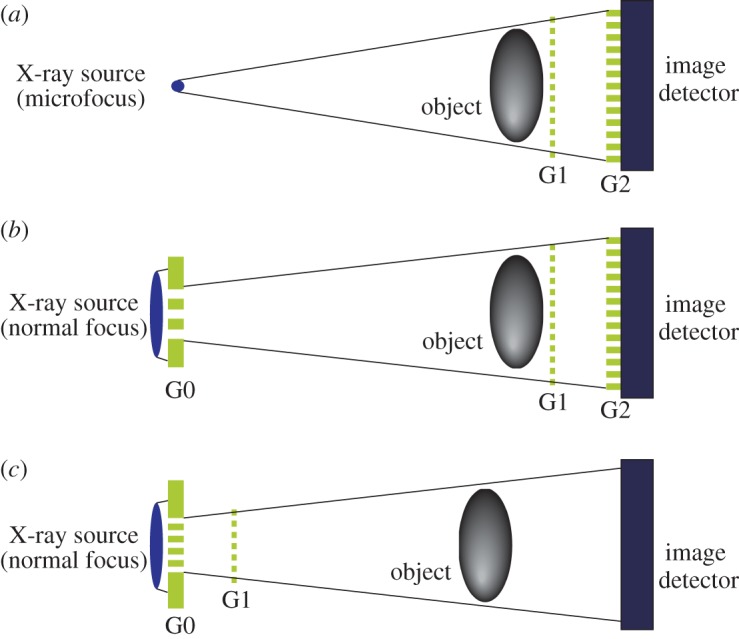
Configurations of X-ray grating interferometry, where transmission gratings (G0, G1 and G2) are arranged in line along the X-ray axis. (*a*) X-ray Talbot interferometer, (*b*) X-ray Talbot–Lau interferometer and (*c*) X-ray Lau interferometer. (Online version in colour.)

The configurations of grating interferometry are shown in figure 1. An X-ray Talbot interferometer consisting of a phase grating (G1) and an amplitude grating (G2), shown in figure 1*a*, functions with X-rays of a spatial coherence length comparable to or larger than the grating pitch, which is of the order of micrometres. A microfocus X-ray generator meets this demand. However, especially for clinical use, the flux from a microfocus X-ray generator is insufficient because the resultant exposure time is too long for patients. An X-ray Talbot–Lau interferometer, shown in figure 1*b*, brought a breakthrough because a normal-focus X-ray generator that is used in hospitals routinely is available. Another amplitude grating (G0) is added to the configuration of the Talbot interferometer to filter out some of the X-rays so that the contrast generated downstream by G1 and G2 does not disappear.

In order to realize an X-ray Talbot (Talbot–Lau) interferometer, the fabrication of G2, which is an X-ray amplitude grating, is a key issue, because a high-aspect-ratio pattern is required with a large area. G1 is normally a phase grating and fabricated comparatively easily.

Figure 1*c* is a relatively new idea, where G1 is shifted towards G0 and the use of G2 is omitted [7]. Provided that G0 of a pitch of about 1 μm is fabricated, this approach may be practicable in the future.

In this paper, we introduce a project for developing machines for clinical use in hospitals based on X-ray Talbot–Lau interferometry shown in figure 1*b*.

## 2. Development for clinical diagnosis

### (a) Targets

We started on the development by fabricating especially large gratings, the area of which determines the field of view of X-ray phase imaging. In order to realize a large field of view, scanning an object across a given field of view may be another possible approach. However, radiologists in clinics strongly prefer to have an image without the scanning procedure for practical reasons, for example an increase in examination time.

As the fabrication of a large-area and high-aspect-ratio line-and-space grating pattern is not easy, its clinical application to diagnosis attainable with a comparatively small field of view should be considered first. Therefore, we selected two targets: (i) diagnoses of joint diseases, for example articular rheumatism, and (ii) diagnosis of breast cancer (mammography). The former relies on the sensitivity to cartilage by X-ray Talbot–Lau interferometry, and fingers or a palm are assumed to be imaged with a field of view of 50×50 mm. For the latter, to fit the size of a whole breast, a larger field of view should be created. Because we could fabricate a 100×100 mm grating, we set mammography application as the second step.

### (b) Grating fabrication and system design

To make the body size of a machine so compact as to be used routinely in hospitals, the pitch of gratings is calculated to be of the order of micrometres, enabling a system including an X-ray generator with a total size of about 2 m. At the same time, as the amplitude grating must block hard X-rays, the height of the line along the X-ray path must be as large as possible. Even when a heavy metal like gold is used, a height of several tens of micrometres is required. Such a pattern should be formed with a large area to create a wide field of view for X-ray phase imaging, as mentioned.

We developed an amplitude grating for G2 with the technique of X-ray lithography at the NewSUBARU synchrotron facility, Japan, and gold electroplating. A line-and-space pattern was formed by X-ray lithography, and gold electroplating was performed between the resultant X-ray resist lines. Consequently, a grating whose pitch, height and area were 5.3 μm, 30 μm and 100×100 mm, respectively, could be developed [8]. If the area can be compromised to be 60×60 mm, the height can be increased successfully to about 50 μm. The resultant grating was not completely uniform because of local defects and pattern deformation. The latter is considered to be caused by the deformation of the X-ray mask used for X-ray lithography and local defects. However, X-ray phase imaging results are not seriously affected because the built-in contrast produced by the defects and pattern deformation can be subtracted from the image with the data obtained without an object.

For G1, a phase grating with gold stripes was fabricated. The height of the stripes was selected to produce *π*/2 phase shift for 28 keV X-rays. Some literature has reported a grating-interferometry configuration where a *π* phase grating was used for G1 [5,9]. In that case, the pitch of G2 should be half. For a clinical machine that assumes the use of X-rays of a continuous spectrum (bremsstrahlung), we believe that a *π*/2 phase grating is suitable. Given the structure of a phase grating, the produced phase shift is inversely proportional to the X-ray energy. When a *π* phase grating is illuminated by continuous-spectrum X-rays, a self-image corresponding to the G2 pitch is produced at a specific range of spectrum. X-rays outside of this range produce a self-image of a doubled period as with a *π*/2 phase grating and do not contribute to phase contrast formation. When a *π*/2 phase grating is used, the period of the self-image is constant within the spectrum range of bremsstrahlung reaching an image detector. Therefore, X-rays are used more effectively for X-ray phase imaging.

### (c) Dose

The high sensitivity of X-ray phase imaging may contribute to reducing the X-ray dose. This is true when an object observable by the conventional X-ray method, for example a bone, is examined and compared. However, we aim at depicting structures undetectable by conventional X-ray imaging. Therefore, the targets described above are set to be approached with a dose acceptable to patients. Although we consider the balance between the gain in the sensitivity and dose reduction, diagnosis performed by conventional machines, for example normal bone fracture, is not within the scope of this development.

After the preliminary study on the sensitivity to cartilage [10], we found that several milligray, which are comparable to or somewhat larger than that used by conventional mammography, were necessary to depict cartilage with a satisfactory signal-to-noise ratio. Therefore, we started the design and development of a prototype to depict cartilage in human fingers with a skin dose below 10 mGy and as low as possible.

## 3. Pilot operation in hospital

**Figure 2.**
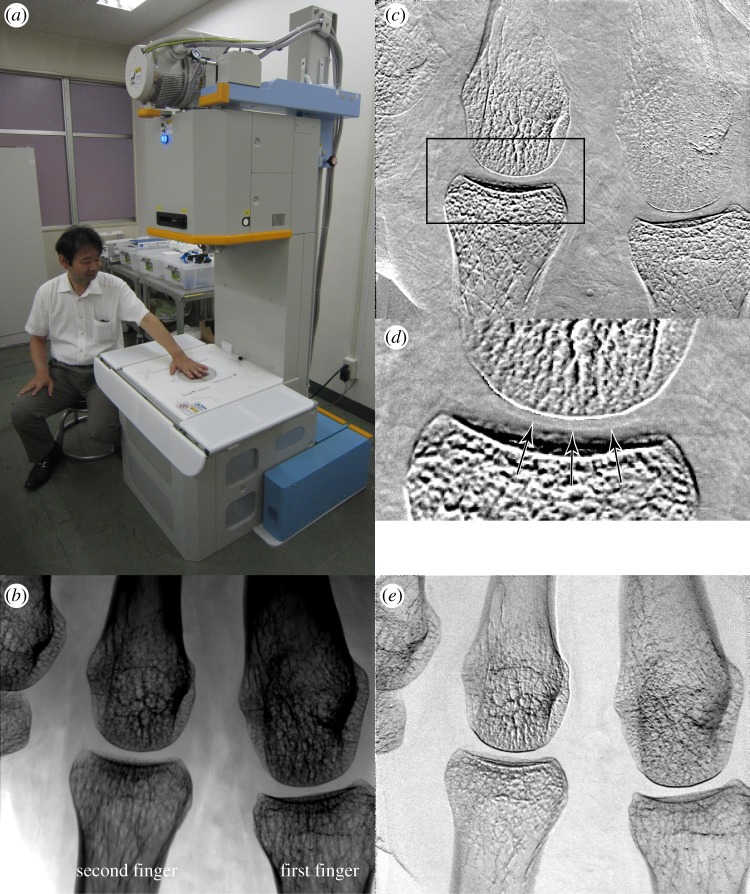
(*a*) An X-ray Talbot–Lau phase imaging system installed in a hospital, and resultant (*b*) absorption image, (*c*) differential phase image, (*d*) its zoomed image at the rectangle indicated in (*c*), and (*e*) visibility image of a part of the first author's palm. X-ray refraction in the vertical direction of the images is sensed. Cartilage in a joint is revealed as indicated by the arrows in (*d*). The blurry feature in the upper right of (*c*) is considered to be due to movement during the measurement. (Online version in colour.)

In the previous study, a machine with a horizontal configuration was used for the convenience of experiments; that is, an X-ray source, gratings, an object to be inspected and an image detector were aligned by stages on horizontal guide rails [10]. For clinical use, however, a vertical configuration is occasionally preferable because a part of a body (e.g. palm) can be placed and held on a table easily. Therefore, as shown in figure 2*a*, a standing-type machine has been developed for the study with healthy volunteers and patients in hospital.

The pitches of G0, G1 and G2 were 22.8, 4.3 and 5.3 μm, respectively, and they were aligned so that 28 keV X-rays are optimal. An X-ray tube with a tungsten target of a 0.45 mm focus was located on the top of the machine and operated with a tube voltage of 40 kV_p_ and a tube current of 100 mA. The tube voltage was selected so that the average X-ray energy in the spectrum from the tube was about 28 keV. The size of G2 was 60×60 mm and the effective field of view was 49×49 mm taking into account the magnification of the image. A flat-panel detector, whose pixel size was 85 μm, was located below G2. More detailed description is given in the previous report [11].

Figure 2*b*–*e* shows the result obtained for the palm of a healthy volunteer, who is the first author. The images were generated from a three-step fringe-scanning dataset. The palm was held on a table, beneath which G1 was located, for 32 s, and actual exposure time was 19 s, applying a total skin dose of 5 mGy. Metacarpophalangeal joints of the first and second fingers are in the field of view, and the smooth surface of cartilage is clearly depicted in the differential phase image (figure 2*c*) and its zoomed image (figure 2*d*), as indicated by the arrows. The line-and-space pattern of the gratings was oriented so that the cartilage in the finger joints is effectively depicted in the differential phase image; that is, in figure 2*c*,*d*, X-ray refraction in the vertical direction is sensed. When a disturbed outline of cartilage is observed, the joint would be diagnosed with a suspected disease.

This machine was used for a study with more than 10 healthy volunteers, and the research has shifted to the test with patients.

## 4. Prospect and conclusion

We selected the palm (fingers) and breast as imaging targets for the initial clinical application of X-ray phase imaging. The machine for joint diagnosis is now in operation for patients, and another similar machine aiming at mammography will be in operation in the near future. We expect that the configuration of the developed machine will be acceptable for the first clinical product based on X-ray phase imaging.

A protocol for diagnosis with the ternary images is a crucial subject to be established because not all radiologists and medical doctors are familiar with the contrasts. While the cartilage was depicted in the differential phase image, the visibility image was not effective. However, in mammographic application, the visibility image carries plenty of information [12]. Thus, it is important to make a guideline for diagnosis after understanding the relation between the information extracted by X-ray phase imaging and diseases, preparing a sophisticated procedure for analysing the ternary images jointly.

Along with this main stream of our development, however, we need to improve the performance of the machine. For example, larger gratings are necessary to expand the field of view, enabling applications to other parts of the body. Then, because a thicker part must be imaged, the use of higher-energy X-rays is required by increasing the tube voltage. This is also effective for reducing the X-ray dose. For this purpose, an amplitude grating of a higher stripe pattern is necessary, because the height limits the maximum X-ray energy available in X-ray Talbot–Lau interferometry. Although the fabrication of larger and higher aspect-ratio gratings is a challenging task, such gratings will promote X-ray phase imaging considerably.

Note that the machine was developed for diagnosis for a two-dimensional image. Because multiple structures located along the X-ray axis are overlaid in a resultant image and cannot be differentiated, appending a depth resolution will be required next. Implementation of the concept of X-ray phase imaging into X-ray computed tomography (CT) is possible, allowing us to acquire three-dimensional information. As mentioned, however, the exposure time for obtaining an X-ray differential image with our current configuration is not at a level compatible with clinical X-ray CT scanners. Therefore, reducing the measurement time is crucial for future development. A brighter X-ray source is preferable, but it should be so compact as to be used in a gantry. We can find another clue in the design and alignment of gratings that makes the entire size of the machine smaller than the current size (2 m), because X-ray flux increases in inverse proportion to the square of the distance between the X-ray source and the detector. Note that a CT scanner for a small animal is under development [13]. For clinical applications, a stereoscopic or tomosynthetic approach would be studied first to gain the depth resolution to some extent.

The X-ray phase imaging concept initially developed at synchrotron facilities is thus being translated into hospitals. After an extensive stack of imaging results by radiologists at hospitals, the machine would be used widely for real medicine in the near future. The achievement would rouse subsequent developments for other clinical usages of X-ray phase imaging based on X-ray grating interferometry.
